# One Size Does Not Fit All: In Support of Psychotherapy for Gender Dysphoria

**DOI:** 10.1007/s10508-020-01844-2

**Published:** 2020-10-21

**Authors:** Roberto D’Angelo, Ema Syrulnik, Sasha Ayad, Lisa Marchiano, Dianna Theadora Kenny, Patrick Clarke

**Affiliations:** 1 Institute of Contemporary Psychoanalysis, Los Angeles, CA 90064 USA; 2 Society for Evidence-Based Gender Medicine, Twin Falls, ID USA

Turban, Beckwith, Reisner, and Keuroghlian (2020) published a study in which they set out to examine the effects of gender identity conversion on the mental health of transgender-identifying individuals. Using the data from the 2015 U.S. Transgender Survey (USTS) (James et al., 2016), they found that survey participants who responded affirmatively to the survey question, “Did any professional (such as a psychologist, counselor, religious advisor) try to make you identify only with your sex assigned at birth (in other words, try to stop you being trans)?” reported poorer mental health than those who responded negatively to the question. From this, Turban et al. concluded that gender identity conversion efforts (GICE) are detrimental to mental health and should be avoided in children, adolescents, and adults. The study’s conclusions were widely publicized by mass media outlets to advocate for legislative bans on GICE, with the study authors endorsing these calls (Bever, 2019; Fitzsimons, 2019; Turban & Keuroghlian, 2019).

We agree with Turban et al.’s (2020) position that therapies using coercive tactics to force a change in gender identity have no place in health care. We do, however, take issue with their problematic analysis and their flawed conclusions, which they use to justify the misguided notion that anything other than “affirmative” psychotherapy for gender dysphoria (GD) is harmful and should be banned. Their analysis is compromised by serious methodological flaws, including the use of a biased data sample, reliance on survey questions with poor validity, and the omission of a key control variable, namely subjects’ baseline mental health status. Further, their conclusions are not supported by their own analysis. While they claim to have found evidence that GICE is associated with psychological distress, what they actually found was that those recalling GICE were more likely to be suffering from serious mental illness. Further, Turban et al.’s choice to interpret the said association as evidence of harms of GICE disregards the fact that neither the presence nor the direction of causation can be discerned from this study due to its cross-sectional design. In fact, an alternative explanation for the found association—that individuals with poor underlying mental health were less likely to be affirmed by their therapist as transgender—is just as likely, based on the data presented.

Arguably, even more problematic than the flawed analysis itself is the simplistic “affirmation” versus “conversion” binary, which permeates Turban et al.’s (2020) narrative and establishes the foundation for their analysis and conclusions. The notion that all therapy interventions for GD can be categorically classified into this simplistic binary betrays a misunderstanding of the complexity of psychotherapy. At best, this blunt classification overlooks a wide range of ethical and essential forms of agenda-free psychotherapy that do not fit into such a binary; at worst, it effectively mis-categorizes ethical psychotherapies that do not fit the “affirmation” descriptor as conversion therapies. Stigmatizing non-“affirmative” psychotherapy for GD as “conversion” will reduce access to treatment alternatives for patients seeking non-biomedical solutions to their distress.

We originally raised our concerns about the quality of Turban et al.’s (2020) study and the validity of their conclusions in a Letter to the Editor of *JAMA Psychiatry*, where the study had been published. However, our letter was rejected, apparently due to space limitations. In the ensuing months, as we observed Turban et al.’s unsupported claims of the harms of psychotherapy for GD taking root globally (United Nations, 2020), we felt compelled to write a more detailed critique of the study, which we present here. Our aim is to put the spotlight on the more problematic areas of Turban et al.’s analysis and to illustrate how heeding their recommendations will limit access to ethical psychotherapy for individuals suffering from GD, further disadvantaging this already highly vulnerable population.

## Biased Sample

Turban et al.’s (2020) analysis used data from the 2015 USTS survey of transgender-identifying individuals (James et al., 2016). This survey used convenience sampling, a methodology which generates low-quality data (Bornstein, Jager, & Putnick, 2013). Specifically, the participants were recruited through transgender advocacy organizations and subjects were asked to “pledge” to promote the survey among friends and family. This recruiting method yielded a large but highly skewed sample. While Turban et al. acknowledged that the USTS may not be representative of the U.S. transgender population, they treat it as a valid source of data for major policy recommendations, disregarding the significant bias in the underlying data.

To demonstrate this apparent bias, we have constructed Table 1, which compares the demographic characteristics of the USTS participants to those of transgender participants from a high-quality probability sample collected by the Centers for Disease Control Behavioral Risk Factors Surveillance System (BRFSS) (Baker, 2019; CDC, 2014–2017). As Table 1 illustrates, even after applying weighting to correct for known survey biases, the USTS participants were far more likely to be young (42% vs. 22% were 18–24 years old) and educated (47% vs. 14% had completed post-secondary education) than BRFSS participants. They were far less likely to own a home (16% vs. 55%) or to be married or coupled (18% vs. 46%). They were also much more likely to have a non-binary identity (38% vs. 22%) and a markedly different self-reported sexual orientation: Only 15% of the USTS participants reported a heterosexual orientation, compared to 69% of the BRFSS participants. (It is not clear if sexuality in either case was reported relative to one’s sex or gender identity.)

**Table 1:** Comparison of demographic characteristics of transgender-identifying individuals in the 2015 US Transgender Survey (USTS) and the Behavioral Risk Factor Surveillance System Survey (BRFSS) 2014–2017

	USTS, 2015^a^ Transgender (*n* = 27,715)	BRFSS, 2014–2017^b^ Transgender (*n* = 3075)
Characteristic		
Gender identity		
Transgender women (male to female)	33%	48%^e^
Transgender men (female to male)	29%	30%^e^
Non-binary/gender-non-conforming	38%	22%^e^
Sexual orientation^c^		
Heterosexual	15%	69%
Lesbian or gay	16%	10%
Bisexual	14%	15%
Other^d^	55%	7%
Age		
18–24	42%	22%
25–44	42%	30%
45–64	14%	32%
65 +	2%	17%
Race/ethnicity		
White, non-Hispanic	62%	55%
Black, non-Hispanic	13%	16%
Asian, Native Hawaiian, or Pacific Islander	5%	5%
Other, non-Hispanic	3%	5%
Hispanic	17%	19%
Education level		
Did not graduate high school	2%	21%
Graduated high school	11%	33%
Some college or technical school	40%	32%
Graduated college or technical school	47%	14%
Annual household income		
< 25,000	38%	39%
25,000–49,999	24%	24%
50,000 +	38%	37%
Home ownership		
Own	16%	55%
Rent	44%	35%
Other arrangement	40%	10%
Marital status		
Married or coupled	18%	46%
Divorced, separated, or widowed	10%	21%
Never married	72%	33%

^a^US Transgender Survey, 2015 (James et al., 2016). Weighted data

^b^CDC BRFSS Survey, 2014–2017 (Baker, 2019). Weighted data

^c^Sexual orientation reported based on the respondent self-identification

^d^Combines all the response options other than “homosexual,” “lesbian/gay,” or “bisexual.”

^e^Calculated using 2014–2017 BRFSS data (CDC, 2014–2017). Weighted data

A number of additional data irregularities in the USTS raise further questions about the quality of data captured by the survey. A very high number of the survey participants (nearly 40%) had not transitioned medically or socially at the time of the survey, and a significant number reported no intention to transition in the future. The information about treatments received does not appear to be accurate, as a number of respondents reported the initiation of puberty blockers after the age of 18 years, which is highly improbable (Biggs, 2020). Further, the survey had to develop special weighting due to the unexpectedly high proportion of respondents who reported that they were exactly 18 years old. These irregularities raise serious questions about the reliability of the USTS data.

In addition to these demonstrable data problems, there are a number of other biases in the USTS data that likely skewed the responses. By targeting transgender advocacy groups, the survey underrepresented the experiences of transgender individuals who are not politically engaged. The emphasis on the survey’s goals to highlight the injustices suffered by transgender people during the recruitment stage and in the introduction of the survey instrument itself made it vulnerable to overreporting of adverse experiences due to “demand bias” (also known as the “good subject effect”). This form of bias occurs when the researchers reveal their hypothesis and aims, which encourages participants to support the investigator’s aims with their answers (Nichols & Maner, 2008; Orne, 1962; Weber & Cook, 1972). Finally, the experiences of detransitioners and desisters were not included, as they were disqualified from completing the survey. Failure to include detransitioned and desisted individuals in research regarding psychological interventions for GD is a serious oversight. These individuals, whose transgender identification was transient, may have been hurt by therapies that affirmed them as transgender, and may have benefitted from therapies that helped them successfully ameliorate their GD (D’Angelo, 2020b).

These serious limitations of the USTS survey greatly undermine the validity of the findings it produced. It is imperative that any analysis based on this low-quality biased sample is validated using a high-quality probability sample before any recommendations stemming from the analysis of these data can be used to shape clinical or policy decisions.

## Invalid Measure of Gender Conversion Therapy

Turban et al.’s (2020) conclusions rest on the assumption that they have a valid way of determining whether or not a respondent was exposed to the unethical practice of conversion therapy. Yet, the USTS question they relied on (Question 13.2) is too non-specific to serve as a valid measure of gender conversion therapy. Firstly, the question conflates mental health encounters with interactions with other types of professionals. Secondly, there is no information about whether the recalled encounter was self-initiated or coerced. Thirdly, it does not differentiate between diagnostic evaluations or a specific therapeutic intervention. There is also no information about whether the focus of the encounter was gender dysphoria or another condition. And finally, it does not determine whether shaming, threats, or other unethical tactics were utilized during the encounter. This lack of context and detail renders the question incapable of differentiating between ethical non-affirmative (neutral) encounters and unethical conversion therapy.

Consider a common situation where the patient is seeking approval for medical treatment for GD, where the role of the therapist is to assess the individual’s mental health to ensure that GD is not secondary to another condition. Such encounters can be experienced by patients as an attempt to withhold the treatment they so desperately want (Chiland, 1997). Further, patients with psychiatric diagnoses, highly prevalent in transgender-identifying populations (Gijs, van der Putten-Bierman, & De Cuypere, 2013; Goodman & Nash, 2018; Wanta, Niforatos, Durbak, Viguera, & Altinay, 2019), can potentially experience or misinterpret neutral interpersonal interactions as invalidating or rejecting (Barnow et al., 2009; Beck & Bredemeier, 2016; Gotlib, 1983). Not only does the survey question provide no detail to help discriminate between these essential therapy encounters and unethical conversion therapy, but it arguably biases the recall of neutral encounters toward recall of conversion by using emotionally charged language (e.g., “stop you being trans”) and by conflating recall of religiously motivated encounters with clinical ones.

Turban et al. (2020) ignored these issues and instead created a veneer of certainty by referring to USTS question 13.2 as GICE and used it throughout the paper as though it were a valid equivalent of conversion therapy. Not only it the term itself novel (the lead author referred to the same USTS question by yet another term, “PACGI,” in a publication just weeks earlier [Turban, King, Reisner, & Keuroghlian, 2019]), but its equivalency to conversion therapy is highly debatable, in part due to the fact that the term itself has not been defined, other than through a circular reference to USTS question 13.2 itself.^1^ Accounting for the many gray areas in the question wording, we propose that GICE is “any professional encounter which the subject recalls as non-affirmative of their transgender identity.” As we have demonstrated, it is not uncommon for agenda-free, neutral therapy interventions to be experienced by the subjects as non-affirmative. However, non-affirmative is not the same as “conversion,” as the latter implies a therapist agenda and an aim for a fixed outcome (American Psychological Association, 2015). In fact, it is the utter inability of USTS question 13.2, and consequently, GICE, to differentiate between agenda-free ethical psychotherapy and coercive, agenda-driven therapy, that is the Achilles heel of Turban et al.’s entire argument.

## Misinterpretation of a Key Scale

A key finding of Turban et al.’s (2020) analysis is that the USTS participants who recalled exposure to GICE were more likely to report severe psychological distress, as evidenced by their score of $\ge$ 13 on the K-6 scale. From this, Turban et al. concluded that GICE has adverse effects on mental health. We will address the unsupported claim of causation in a subsequent section. Here, we would like to further explore the use of the K-6 scale to make these claims, and its implications.

The K-6 scale, and its cutoff score of $\ge$ 13, was specifically developed by Kessler et al. (2003) in order to discriminate between cases of non-specific psychological distress and cases of serious mental illness (SMI). Scoring $\ge$ 13 is predictive of having a DSM diagnosis of schizophrenia, bipolar disorder, and a range of other major mental health conditions that cause serious functional impairment (Substance Abuse and Mental Health Services Administration, 2020). Thus, Turban et al.’s (2020) finding of an association between the recall of GICE and scoring $\ge$ 13 actually suggests that the USTS participants recalling GICE were more likely to have a severe mental illnesses diagnosis than those not recalling GICE. Further, any claim of causation, which Turban et al. continue to suggest throughout the paper (however unsupported by the study design), would imply that exposure to GICE caused serious mental illness, in previously mentally well populations. This is a highly speculative and implausible hypothesis, which further challenges their claims.

## Omission of a Key Control Variable

Turban et al.’s (2020) hypothesis, namely, that GICE exposure (during lifetime, as well as in childhood) causes poor mental health and contributes to suicide attempts, is further weakened by a significant flaw in their data analysis: failure to control for the individuals’ pre-GICE-exposure mental health status. Not only does this critical omission confound the association between exposure to GICE and present mental health, but it may mask reverse causation, namely, that it was the individual’s underlying poor mental health that led to their experience of GICE in the first place.

Let us revisit the example of a common clinical encounter in which a person with GD and one or more comorbid psychiatric conditions presents for assessment with the goal of obtaining approval for cross-sex hormones. An assessment of such a complex presentation generally requires multiple sessions and involves ascertaining whether the GD is secondary to another condition. It is also likely that the clinician might focus on treating the comorbid condition(s) first, before pursuing “gender-affirming” interventions. While such a contact would be recalled by the respondent as non-affirmative and thus likely classified as GICE, it is the patient’s poor mental health status that led to the non-affirming content of the encounter, rather than vice versa. If the said individual had attempted suicide in the past or continued to struggle with mental illness more recently, Turban et al.’s (2020) analysis would erroneously conclude that GICE was likely responsible for those difficulties, when, in fact, no such causation occurred.

In fact, failure to control for the subjects’ baseline mental health makes it impossible to determine whether the mental health or the suicidality of subjects worsened, stayed the same, or potentially even improved after the non-affirming encounter. Given the high rate of co-occurring mental illness in transgender-identifying patients (Gijs et al., 2013; Goodman & Nash, 2018; Wanta et al., 2019), failure to control for prior mental health status is a serious methodological flaw.

## Internal Inconsistencies in Mental Health Measures

Turban et al.’s (2020) finding that mental health outcomes of persons exposed to GICE are worse than those whose encounters were “gender-affirming” is weakened by internal inconsistencies in the mental health outcome measures. We have already discussed the fact that the threshold chosen by Turban et al. on the K-6 scale detects serious mental illness, rather than distress. Another measure of psychological distress chosen by Turban et al.—substance misuse—was not significantly different between GICE and the non-GICE group. More importantly, there is a lack of consistency in the suicide measures. While lifetime suicide attempts were elevated among the GICE group, total suicide attempts in the prior 12 months, as well as suicide attempts requiring hospitalization, which generally indicate more serious attempts rather than non-suicidal self-injury, were not significantly different between the two groups. Turban et al. did not address this inconsistency. Nor did they explore the relationship between suicidality and the higher levels of serious mental illness among the GICE group, despite the well-documented link between serious mental illness and suicide (Bertolote, Fleischmann, De Leo, & Wasserman, 2004). Turban et al. did not heed their own warning not to attribute the increased lifetime suicidality entirely to GICE since “other factors are also likely to be associated with suicidality among gender-diverse people.” Instead, they treat the inconsistent and unclear association between GICE and suicidality as causative and infuse it with an air of certainty by elevating it into title of their paper.

## Claim of Causation When Only an Association Has Been Found

Although a causative relationship between recalled GICE and adverse mental health status is possible (even if direction of the causality is unclear), the cross-sectional design of the USTS is not capable of determining causation. While Turban et al. (2020) acknowledged this limitation and correctly referred to the relationship they found as an association, they strongly implied causation throughout their discussion, as well as in their “Conclusions and Relevance” section, which states, “These results support policy statements from several professional organizations that have discouraged this [GICE] practice.” Presenting a highly confounded association as causation is a serious error, given its potential to dangerously misinform and mislead clinicians, policymakers, and the public at large about this important issue.

## Discussion

The fact that coercive techniques to force unwanted changes in individuals are unethical and have no place is modern psychotherapy is self-evident and needs no additional justification. However, as we have demonstrated, Turban et al. (2020) failed to prove that GICE, as defined by affirmative answers to the USTS question, caused poor mental health or suicide attempts in study subjects. Further, since Turban et al. failed to establish equivalence between GICE, which likely subsumes a range of ethical non-affirmative interventions, and “gender conversion therapy,” which implies unethical and coercive attempts to force a change in one’s identity, their use of the study findings in support of a ban on “gender conversion therapy” is without any foundation.

Rather than appropriately acknowledging the significant study limitations and calling for more research, Turban et al. (2020) used their flawed findings to engage in a media campaign promoting legislative bans of GICE. Two of the study authors penned an op-ed in which they state, “It’s time for conversion efforts to be illegal in every state, before more people die” (Turban & Keuroghlian, 2019). Turban, the lead author, repeated these sweeping, emotive claims on several highly visible national media platforms (Bever, 2019; Fitzsimons, 2019). In contrast, the debate regarding this study in the scientific arena was not allowed to occur. To the best of our knowledge, all of the letters written to the Editor of *JAMA Psychiatry*, many by respected academics and clinicians who outlined the serious problems in the study, have been rejected (some of them were later submitted as non-indexed comments in the online publication). The omission of these important arguments from the scientific discourse stifles scientific debate and perpetuates the current politicization of transgender health care, where treatment decisions are increasingly legislated by politicians.

While the poor study methodology is unfortunate, arguably, the most problematic aspect of Turban et al.’s (2020) work is the choice to view psychotherapy through a binary of “affirmation” versus “conversion,” resulting in a conflation of ethical non-affirmative psychotherapy with conversion therapy. The self-evident crudeness of the GICE versus “affirmation” binary, promoted by Turban et al., and the potential harms of such a simplistic view of psychotherapy are illustrated by the following examples.

Consider a female victim of sexual assault, who subsequently develops an intense discomfort with her female anatomy and expresses a desire to undergo biomedical interventions to change her body. It would be unethical for the clinician to overlook the contribution of sexual victimization to this nascent GD. A therapist enthusiastically supporting this patient’s new male identity would be failing to provide appropriate treatment for what amounts to a post-traumatic condition, instead providing an inappropriate treatment with the potential to harm. Similarly, a boy who has been traumatized by relentless bullying due to his gender “non-conformity” (e.g., interest in classical music or fashion and avoidance of sports) may conclude that if he were a girl then he would “fit in” and the humiliation would stop. In this case too, gender-affirming interventions miss the mark when what this traumatized young person requires is psychotherapy.

Another obvious difficulty arises when same-sex attracted adolescents report cross-sex identifications. Research shows that a high number of homosexual adults have experienced periods of “cross-sex” behaviors and cross-gender identification in childhood and adolescence, often to a degree that is severe enough to warrant the diagnosis of GD, or gender identity disorder, as it was previously known (Bailey & Zucker, 1995; Bell, Weinberg, & Hammersmith, 1981; Hiestand & Levitt, 2005; Li, Kung, & Hines, 2017). When a dysphoric same-sex attracted young person in the midst of this developmental process presents for mental health care, a clinician overtly affirming the patient’s cross-sex gender identity would be failing this patient by not addressing the patient’s struggle with same-sex attraction and/or internalized homophobia. In fact, some homophobic societies and indeed families that reject homosexuality among their children have embraced the “affirmative” biomedical pathway (Bannerman, 2020; Hamedani, 2014), which poses a question as to whether “affirmative” care in some instances serves the role of gay conversion therapy.

Further, GD can present as a transient symptom that resolves spontaneously or in the context of developmentally informed psychotherapeutic treatment. Some common examples of transient gender-dysphoric states include adolescents girls, often on the autism spectrum, experiencing distress around the physical and social changes of puberty or gender-non-conforming young women struggling with shame about being seen as “butch.” These individuals, searching for ways to understand and remedy their distress, can incorrectly attribute their discomfort to being transgender. Several case reports (Churcher Clarke & Spiliadis, 2019; Lemma, 2018; Spiliadis, 2019) indicate that the distress of young people with GD can lessen or resolve with appropriate psychotherapeutic interventions that address the central issues.

If anything other than “affirmation” is viewed as GICE, it follows that the provision of psychotherapy in these clinical scenarios would be seen as harmful conversion efforts. Yet these therapeutic interventions do not aim to convert or consolidate an identity, but instead aim to help individuals gain a deeper understanding of their discomfort with themselves, the factors that have contributed to their distress, and their motivations for seeking transition (Bonfatto & Crasnow, 2018; D’Angelo 2020a). These exploratory questions are consistent with the principle of therapeutic neutrality—a cornerstone of ethical psychotherapy (Simon, 1992). In fact, both “conversion” and “affirmation” therapy efforts carry the risk of undue influence, potentially compromising patient autonomy. In contrast, the provision of a neutral, unbiased psychotherapeutic process that allows these patients to clarify their feelings and assess the various treatment options, which range from non-invasive to highly invasive, irreversible procedures, is arguably the only way that meaningful informed consent for the latter can be obtained (Levine, 2018).

Turban et al.’s (2020) unproven assertion that non-affirming therapies are dangerous stands in contrast to the documented risks and uncertainties associated with hormonal and surgical interventions that are a core part of the “affirmation” treatment path. Until recently, puberty blockers were considered safe and fully reversible, but there is now emerging evidence of their adverse effects on the bone and brain health (Klink, Caris, Heijboer, van Trotsenburg, & Rotteveel, 2015; Joseph, Ting, & Butler, 2019; Schneider et al., 2017). Additionally, since almost all of the children treated with puberty blockers proceed to cross-sex hormones (de Vries et al., 2014), concerns have been raised that puberty blockers may consolidate gender dysphoria in young people, putting them on a lifelong path of biomedical interventions.

Cross-sex hormones are associated with cardiovascular complications, including a fourfold increased risk of heart attacks in biological females, and a threefold increase in the incidence of venous thromboembolism in biological males (Alzahrani et al., 2019; Nota et al., 2019). “Gender-affirming” surgeries can cause urethral stricture, neo-vaginal stenosis and prolapse, and long-term post-mastectomy pain (Larsson, Ahm Sørensen, & Bille, 2017; Manrique et al., 2018; Rashid and Tamimy, 2013; Santucci, 2018). The effects of “gender-affirmative” care on fertility have not been adequately studied, but infertility is a likely outcome, depending on the specific treatments pursued. It remains unclear whether fertility concerns will be important to this group of patients as they mature, but increasingly, gender centers are recommending fertility preservation procedures prior to undergoing hormonal interventions.

Given the absence of robust long-term evidence that the benefits of biomedical interventions outweigh the potential for harm, especially among young people (Heneghan & Jefferson, 2019), it is self-evident that the least-invasive treatment options should be pursued before progressing to more risky and irreversible interventions. To the extent that psychological treatments can help an individual obtain relief from GD without undergoing body-altering interventions, ensuring access to these interventions is not only ethical and prudent but also essential.

The importance of continued access to non-affirmation–non-conversion, agenda-free evaluation, and treatment is further underscored by the increasing numbers of detransitioning patients speaking out in social media forums following gender transitions they have come to regret (Entwistle, 2020). The rate of regret, detransition, and desistance from transgender identification is largely unknown (Butler & Hutchinson, 2020). The majority of patients with classical, childhood-onset gender dysphoria (61%-98%) desist from transgender identification some time in adolescence or young adulthood (Korte et al., 2008; Steensma, McGuire, Kreukels, Beekman, & Cohen-Kettenis, 2013; Zucker, 2018). The minority who persist with their transgender identification into adulthood and undergo “gender-affirmative” surgeries have been reported to have low rates of regret (van de Grift, Elaut, Cerwenka, Cohen-Kettenis, & Kreukels, 2018) and detransition (Dhejne, Öberg, Arver, & Landén, 2014). However, these studies may understate true regret rates due to overly stringent definitions of regret (i.e., requiring an official application for reversal of the legal gender status), very high rates of participant loss to follow-up (22%-63%) (D’Angelo, 2018), and an unexplored relationship between regret and high rates of post-transition suicide (Dhejne et al., 2011).

The novel cohort of young GD patients increasingly presenting for help is poorly understood. It is overrepresented by adolescent females with recent-onset GD and with comorbid mental health and neurocognitive issues (Bewley, Clifford, McCartney, & Byng, 2019; de Graaf, Giovanardi, Zitz, & Carmichael, 2018; Kaltiala-Heino, Bergman, Työläjärvi, & Frisen, 2018; Littman, 2018; Zucker, 2019). The trajectory of GD among these young patients, including the rates of desistance and detransition, remains unknown. However, many of us, along with our colleagues, are seeing increasing numbers of detransitioners with adolescent-onset GD who regret not having received exploratory psychotherapy to help them understand their distress and the desire to transition before they underwent irreversible medical and surgical treatments. Equally concerning, a number report that when doubts about their own transgender status arose, their therapists continued to affirm them as transgender, attributing their doubts to internalized transphobia, and encouraging them to continue medical interventions, which, in turn, unnecessarily exacerbated the psychological and physical harms.

Advocates of “affirmative care” tend to downplay the risks of iatrogenic harms resulting from inappropriate transitions and minimize the seriousness of the resulting harms by describing them as merely “cosmetic” (Turban & Keuroghlian, 2018). In stark contrast to these assertions, we are seeing increasing numbers of patients who feel deeply traumatized by inappropriate transitions. They suffer from irreversible physical changes, including alterations to their genitals and sexual function, sterility, painful vaginal atrophy, chest/breast alteration and scarring, deepening of the voice, unwanted permanent changes to facial hair growth, male-pattern baldness, urinary incontinence, and other lasting effects. Apart from the distress that these changes cause, they also negatively impact many areas of their lives, including their ability to form a stable gender identity (many feel trapped in a “gender no-man’s land”), to find romantic partners and supportive social networks, to bear children, or to secure employment. The process of coming to terms with these consequences of their transition is psychologically difficult and can be profoundly painful.

Given the risky and irreversible nature of “gender-affirming” treatments, it is concerning that for many years now, there has been a lack of systematic research into the role that developmentally informed psychotherapy can play in the amelioration of GD, especially among young people. The need for the continued development and evaluation of non-invasive psychological treatment alternatives for GD has never been more urgent, given the fact that over 3% of young people report transgender identification or ideation (Johns et al., 2019). Given the sheer magnitude of this change, and the potential for exponential growth in the number of individuals who are medically harmed, it is time to raise the bar on science and to heed the first and most fundamental tenet of medicine: “First, do no harm.”

## Conclusions

Turban et al.’s (2020) singular endorsement of “affirmative” therapies, which their data failed to substantiate, contributes to the alarming trend to frame any non-“affirming” approaches as harmful. We are deeply concerned that this false dichotomy, reinforced by Turban et al.’s unproven claims of the harms of GICE, will have a chilling effect on the ethical psychotherapists’ willingness to take on complex GD patients, which will make it much harder for GD individuals to access quality mental health care. We maintain that availability of a broad range of non-coercive, ethical psychotherapies for individuals with GD is essential to meaningful informed consent, which requires consideration of the full range of treatment options, from highly invasive to non-invasive. Further, given the potential of agenda-free psychotherapy to ameliorate GD non-invasively among young people with GD, withholding this type of intervention, while promoting “affirmation” approaches that pave the way to medical transition, is ethically questionable.

We believe that exploratory psychotherapy that is neither “affirmation” nor “conversion” should be the first-line treatment for all young people with GD, potentially reducing the need for invasive and irreversible medical procedures. This is especially critical now, when we are witnessing an exponential rise in the incidence of young people with GD who have diverse and complex mental health presentations and require careful assessment and treatment planning.

We are concerned about the deficit in our knowledge base about psychological interventions for GD, beyond a few successful but small case studies, and we fear that the erroneous conclusions presented by Turban et al. (2020) will make it less likely that such research will be carried out in the future. We call on the scientific community to resist the stigmatization of psychotherapy for GD and to support rigorous outcome research investigating the effectiveness of various psychological treatments aimed at ameliorating or resolving GD. The outcomes of psychotherapeutic treatments must be compared to those of biomedical interventions, so that evidence-based standards of care that allow patients and clinicians to make fully informed decisions about how best to alleviate GD can be developed and put into practice.

## Footnotes

1 Psychological Attempts to Change Gender Identity.
